# Supplementary material for: Insights on the Excitation Spectrum of Graphene Contacted with a Pt Skin
Source: Nanomaterials (Basel). 2020 Apr 8;10(4):703. doi: 10.3390/nano10040703 (PMC7221714; doi:10.3390/nano10040703)
Supplement: Supplementary file 1 [file nanomaterials-10-00703-s001.pdf]

nanomaterials

Supporting Information

S1. Graphene growth on Pt-skin-terminated $Pt_3Ni(111)$

To establish the optimal temperature for which graphene phase is formed on the Pt_3Ni alloy, we followed in real time the evolution of the C 1*s* core level as function of the substrate temperature (300-1000 K) in an ethylene environment (Figure S1, panels a-b).

Selected X-ray photoelectron spectroscopy (XPS) spectra in the C 1*s* region acquired at 350, 650, 800 and K are reported in panels c-e of **Error! Reference source not found.**, respectively. At room temperature (RT), the adsorption of ethylene (C_2H_4) on $Pt_3Ni(111)$ is fully dissociative, as evidenced by the absence of the spectral components related to C_2H_4, whose binding energy (BE) is 283.1 eV [1]. Conversely, the C 1s core level measured in the 300-400 K range in the ethylene-dosed surface is dominated by ethylidyne (CCH_3), whose component has BE 284.0 eV.[1] The shoulder at 283.6 eV is ascribed to C-H groups,[1] which have found to be the most stable hydrocarbon fragments on Pt(111)[1].

It is worth noticing that the conversion of ethylene in ethylidyne on the Pt(111) surface begins at 255 K[2]. However, at RT well-defined islands of unreacted ethylene have been imaged by scanning tunnelling microscopy[3], while the complete conversion occurs only at 320 K, as shown by XPS experiments by Fuhrmann et al. [1].

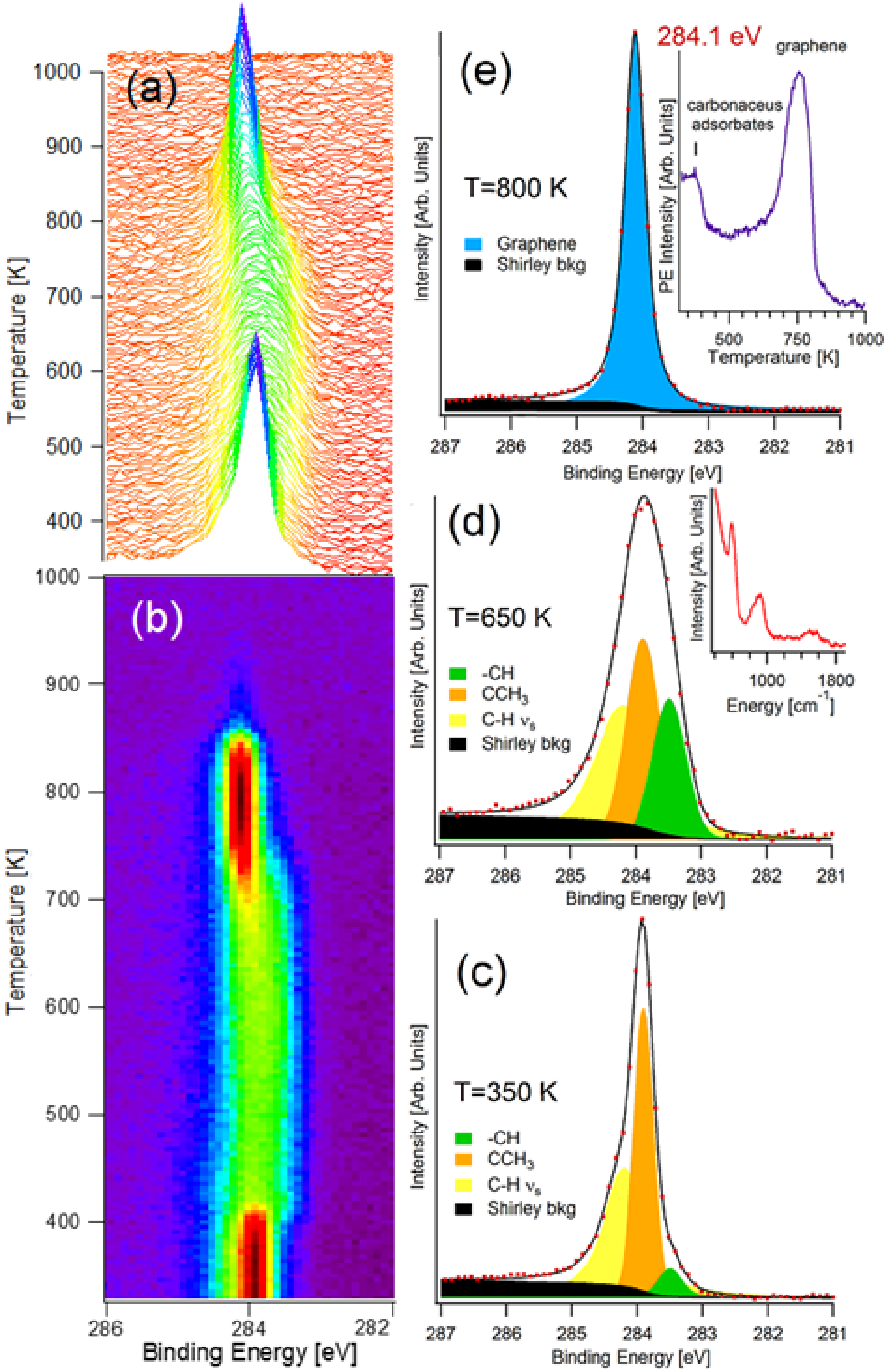

Figure S1. Panels (a) and (b) report the real-time evolution of the C 1s signal during the heating of the $Pt_3Ni(111)$ in an ethylene environment. Panels (c), (d), and (e) represent the C 1s core level measured with the sample kept at 350, 650, and 800 K, respectively. The black regions represent the Shirley background, which has been subtracted before fitting the resulting spectrum with Voigt line-shapes. The inset of panel (d) reports the corresponding vibrational spectrum. The inset of panel (e) shows the behaviour of the photoemission intensity at a BE of 284.1 eV, corresponding to the graphene phase, as a function of temperature.

From the temperature evolution of the C 1*s* core level for ethylene-dosed $Pt_3Ni(111)$ in the range between 438 and 715 K, we can assert that CCH_3 evolves into -CH groups, as evidenced by the relative increase of the component at BE 284.2 eV. The spectral weight of the peak at 283.6 eV changes with temperature and it disappears at 741 K.

The C 1*s* signal for temperature in the range 777 - 823 K has a single narrow component at 284.1 eV. Both the sharpness of the line-shape and its BE suggest that a graphene phase is formed in this temperature range.

However, for temperature higher than 823 K, the intensity of the C 1*s* signal abruptly decreases (**Error! Reference source not found.**, panels a and b) and disappears at 921 K. This finding indicates that carbon atoms dissolve into the bulk of the Pt_3Ni crystal.

Remarkably, we are led to conclude that the presence of Ni atoms in the alloy does not induce any change in the thermodynamics of the nucleation of graphene islands, as instead reported for the Ni/Au alloy [4]. The presence of Ni atoms underneath the Pt skin does not change the temperature of formation of Gr, while it coincides with that of graphene on Pt(111)[5]. On the contrary, in the case of Ni/Au[4], graphene nucleates on the residual Ni patches at the surface and this explains why the growth temperature corresponds to that reported for graphene/Ni(111)[6] and graphene/Ni(110)[7]. Therefore, we can assert that the mere presence of Ni atoms in a crystalline lattice of an alloy does not play any role in lowering the temperature for graphene formation. By contrast, we suggest that the presence of residual Ni nanoplatelets over the surface could catalyze graphene nucleation at a temperature close to that found in Ni single crystals.

It is noteworthy that no temperature-induced segregation of Ni has been observed during time-resolved XPS experiments in the region of Pt 4*f* and Ni 3*p* core levels. In fact, the ratio between Pt 4*f* and Ni 3*p* peaks remains unchanged during the heating.

S2. Orientation of the graphene lattice with respect to the Pt skin

The low-energy electron diffraction (LEED) pattern observed for graphene grown on $Pt_3Ni(111)$ at a fixed temperature of 780 K (top part of Figure S2) exhibits spots from the hexagonal lattice of $Pt_3Ni(111)$ (inner spots) superimposed with semi-arcs arising from diffraction from the graphene overstructure (outer spots). The presence of semi-arcs indicates that differently rotated domains exist in the graphene superlattice. Such a LEED pattern is similar but not identical to that of graphene on Pt(111). In graphene on Pt(111), three domains are predominant: one aligned with the substrate, other ones rotated by ±19° and another one rotated by 30° [5]. The quantitative analysis of the intensity of diffraction spots as a function of the angle I(θ) reveals that, compared to graphene/Pt(111) (Figure S2, middle panel), diffraction peaks in graphene/$Pt_3Ni(111)$ are very broad (Figure S2, bottom panel), as a result of higher disorder in the graphene overlayer. Nevertheless, the analysis of I(θ) indicates that in graphene/$Pt_3Ni(111)$ the same domains observed in graphene/Pt(111) exist, due to the presence of a Pt skin in $Pt_3Ni(111)$ [8-10].

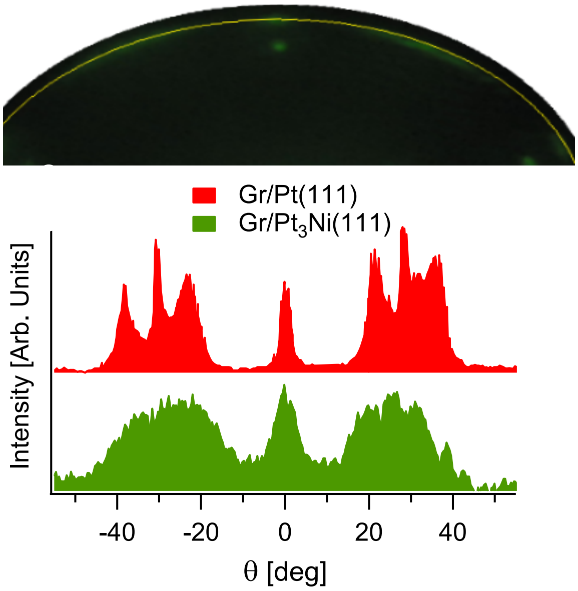

Figure S2. (top panel) LEED pattern of graphene/Pt_3Ni(111), acquired at E_P=74 eV. (bottom panel) Intensity of LEED spots of graphene/Pt_3Ni(111) as a function of the angle I(θ) (green area), calculated along the yellow path indicated in the top panel. The I(θ) behavior for the case of graphene/Pt(111) (red area) is also reported for a comparison.

Therefore, as for graphene/Pt(111) [11, 12] , the graphene overlayer grown on the Pt skin of Pt_3Ni(111) is characterized by a moiré superlattice, originated from the minimization of the absolute value of the strain between the graphene and the Pt skin for the different orientations between both atomic lattices.

S3. Electronic properties of graphene supported by Pt-skin-terminated Pt_3Ni(111)

Both the C 1*s* BE (284.1 eV, Figure S1) and the LEED pattern (Figure S2) indicate that graphene formed on the Pt_3Ni(111) surface is supported by the Pt-skin. In details, the BE of the C 1s resembles that of quasi-freestanding graphene on Pt(111) [13], i.e. 284.1 eV, while for graphene/Ni(111) the C 1*s* has two components at 284.4 and 284.8 eV [6, 14, 15], due to the coexistence of top-fcc and bridge-fcc structures [16].

Near-edge X-ray absorption fine structure (NEXAFS) experiments for graphene/Pt skin are reported in Figure S3 for two geometries, with photon incidence grazing (red curve) and nearly-normal (black curve) with respect to the sample in order to probe the 1*s* → π* (~285 eV) and 1*s* → σ * (291-292 eV) transitions, respectively[17]. The highly dichroic C K-edge signal, due to the inherent nature of graphene band structure, with in-plane σ* and out-of-plane π* states, confirms the excellent quality of the graphene layer on Pt_3Ni(111). The pre-edge shoulder observed at ~284 eV has been revealed in graphene/Pt(111)[18], with a weak feature at 288.1 eV [19]. Features in the region between 1s → π* and 1s → σ* transitions are due to interface states, as observed for graphene/Ni(111)[20]. These excitations correspond to electronic transitions toward the interface state above the Fermi level (around the M-point in the Brillouin zone) originating from the hybridization of C p_z orbitals with Ni *d* bands. The intensity of interface states is reduced in the graphene/Pt_3Ni(111) system, because of the presence of Ni atoms only in the second layer of the Pt_3Ni surface[21]. Moreover, their energy is different with respect to graphene/Ni(111), for which interface states are observed at 286.3 eV [20]. The occurrence of interface states in graphene/Pt_3Ni(111) indicates orbital mixing, which is instead absent in graphene/Pt(111)[18].

The 1*s* → σ* transition is composed of two distinct features at 291.4 eV and 292.4 eV. The sharp peak at 291.4 eV corresponds to the creation of an exciton [22]. Its intensity reflects strong correlation effects of electron–hole pairs within the graphene sheet [23]. The broad peak at 292.6 eV is related to the transition from the C 1*s* level to the relatively nondispersing σ* states at the Γ point of the Brillouin

zone [23, 24]. NEXAFS features related to 1*s* → σ* transitions in graphite and graphene/Pt(111) are quite similar to those of graphene/Pt_3Ni(111), while the two features in strongly interacting graphene/metal interfaces, as graphene/Ni(111), overlap resulting into a broad peak[20].

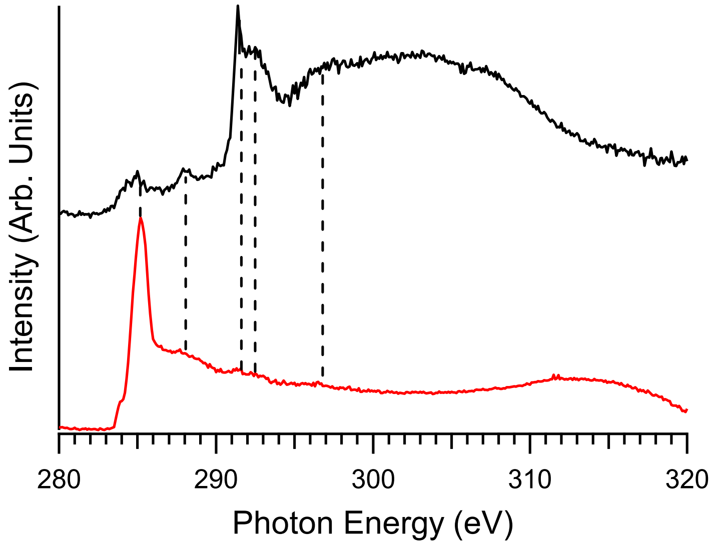

Figure S3. X-ray absorption spectra at the C K-edge measured for graphene/Pt_3Ni(111) at grazing (20°, red curve) and normal incidence (90°, black curve).

Figure S4 shows the angle-resolved photoemission spectroscopy (ARPES) characterization of the grown graphene layer, taken after focusing on a single micrometric graphene domain.

The analysis of ARPES experiments indicates that Gr-derived states around the Fermi level hybridize with 5*d* states of Pt, whose centroid is localized in the nearness of the Fermi level of the Pt_3Ni alloy[25]. On the contrary, the 3*d* band of Ni, located at a BE of ~2 eV[25], is not hybridized with the Dirac cone of the graphene sheet.

Remarkably, we note that the graphene doping results to be 0.0 ± 0.1 eV, i.e. the Fermi level crosses just the Dirac point. Due to the coincidence of the work function of graphene [26] with that of Pt_3Ni(111) [27, 28], graphene/Pt_3Ni(111) forms a charge-neutral graphene/metal contact. By contrast, the graphene sheet on Pt(111) is *p*-type doped by 0.4 eV[29, 30].

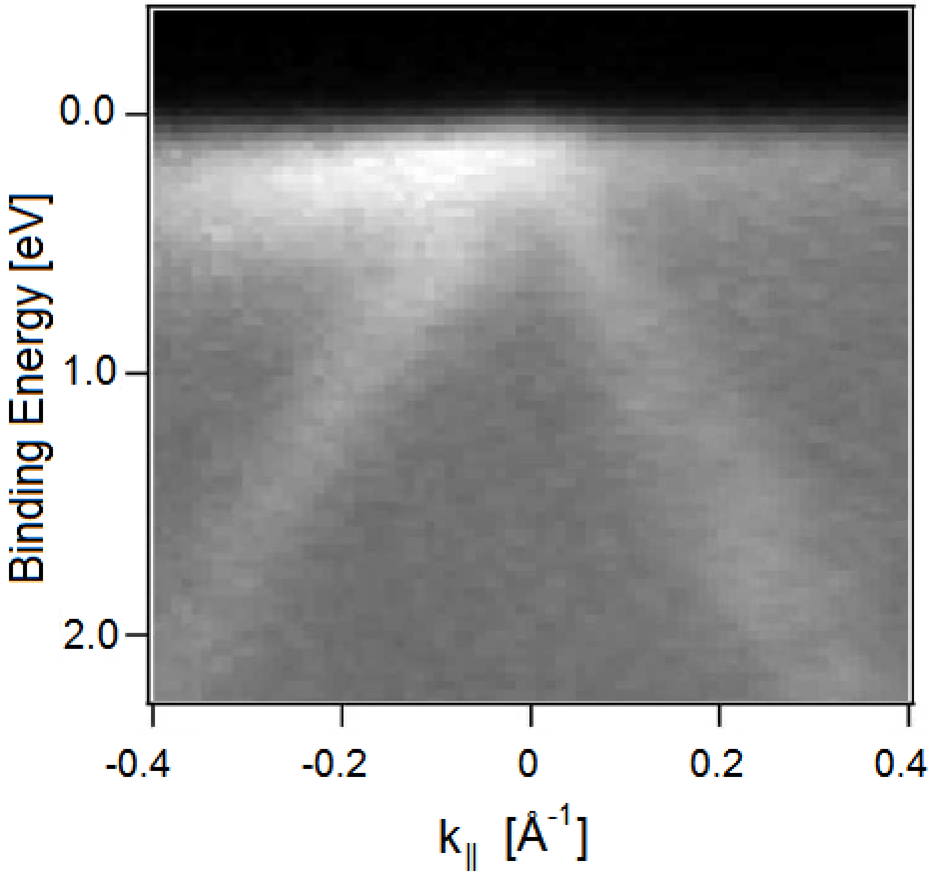

Figure S4. Band structure of graphene/Pt-skin-terminated $Pt_3Ni(111)$ in the nearness of the K point of the Brillouin zone of Gr.

1. Fuhrmann, T.;Kinne, M.;Tränkenschuh, B.;Papp, C.;Zhu, J. F.;Denecke, R.; Steinrück, H. P. Activated adsorption of methane on Pt(111) —an in situ XPS study. *New J. Phys.* **2005**, *7*, 107.
2. Cremer, P.;Stanners, C.;Niemantsverdriet, J. W.;Shen, Y. R.; Somorjai, G. The conversion of di-σ bonded ethylene to ethylidyne on Pt(111) monitored with sum frequency generation: evidence for an ethylidene (or ethyl) intermediate. *Surf. Sci.* **1995**, *328*, 111-118.
3. Land, T. A.;Michely, T.;Behm, R. J.;Hemminger, J. C.; Comsa, G. Direct observation of surface reactions by scanning tunneling microscopy: Ethylene→ethylidyne→carbon particles→graphite on Pt(111). *J. Chem. Phys.* **1992**, *97*, 6774-6783.
4. Weatherup, R. S.;Bayer, B. C.;Blume, R.;Ducati, C.;Baehtz, C.;Schlögl, R.; Hofmann, S. In Situ Characterization of Alloy Catalysts for Low-Temperature Graphene Growth. *Nano Lett.* **2011**, *11*, 4154–4160.
5. Gao, M.;Pan, Y.;Huang, L.;Hu, H.;Zhang, L. Z.;Guo, H. M.;Du, S. X.; Gao, H. J. Epitaxial growth and structural property of graphene on Pt(111). *Appl. Phys. Lett.* **2011**, *98*, 033101.
6. Grüneis, A.;Kummer, K.; Vyalikh, D. V. Dynamics of graphene growth on a metal surface: A time-dependent photoemission study. *New J. Phys.* **2009**, *11*, 073050.
7. Ligato, N.;Cupolillo, A.;Sindona, A.;Riccardi, P.;Pisarra, M.; Caputi, L. S. A comparative study of the plasmonic properties of graphene on lattice-matched and lattice-mismatched Ni surfaces. *Surf. Sci.* **2014**, *626*, 40-43.
8. Stamenković, V. R.;Mun, B. S.;Mayrhofer, K. J. J.;Ross, P. N.; Marković, N. M. Effect of Surface Composition on Electronic Structure, Stability, and Electrocatalytic Properties of Pt-Transition Metal Alloys: Pt-Skin versus Pt-Skeleton Surfaces. *J. Am. Chem. Soc.* **2006**, *128*, 8813-8819.
9. van der Vliet, D. F.;Wang, C.;Li, D.;Paulikas, A. P.;Greeley, J.;Rankin, R. B.;Strmcnik, D.;Tripkovic, D.;Marković, N. M.; Stamenković, V. R. Unique Electrochemical Adsorption Properties of Pt-Skin Surfaces. *Angew. Chem.* **2012**, *124*, 3193–3196.

10. Yang, Z.;Wang, J.; Yu, X. The adsorption, diffusion and dissociation of O_2 on Pt-skin $Pt_3Ni(111)$: A density functional theory study. *Chem. Phys. Lett.* **2010**, *499*, 83-88.
11. Merino, P.;Švec, M.;Pinardi, A. L.;Otero, G.; Martín-Gago, J. A. Strain-Driven Moiré Superstructures of Epitaxial Graphene on Transition Metal Surfaces. *ACS Nano* **2011**, *5*, 5627-5634.
12. Martínez, J. I.;Merino, P.;Pinardi, A. L.;Gonzalo, O.-I.;López, M. F.;Méndez, J.; Martín-Gago, J. A. Role of the Pinning Points in epitaxial Graphene Moiré Superstructures on the Pt (111) Surface. *Sci. Rep.* **2016**, *6*, 20354.
13. Otero, G.;Gonzalez, C.;Pinardi, A. L.;Merino, P.;Gardonio, S.;Lizzit, S.;Blanco-Rey, M.;Van de Ruit, K.;Flipse, C. F. J.;Méndez, J.;de Andrés, P. L.; Martín-Gago, J. A. Ordered Vacancy Network Induced by the Growth of Epitaxial Graphene on Pt(111). *Phys. Rev. Lett.* **2010**, *105*, 216102.
14. Ng, M. L.;Balog, R.;Hornekær, L.;Preobrajenski, A. B.;Vinogradov, N. A.;Mårtensson, N.; Schulte, K. Controlling Hydrogenation of Graphene on Transition Metals. *J. Phys. Chem. C* **2010**, *114*, 18559-18565.
15. Rusz, J.;Preobrajenski, A. B.;Ng, M. L.;Vinogradov, N. A.;Mårtensson, N.;Wessely, O.;Sanyal, B.; Eriksson, O. Dynamical effects in X-ray absorption spectra of graphene and monolayered *h*-BN on Ni(111). *Phys. Rev. B* **2010**, *81*, 073402.
16. Zhao, W.;Kozlov, S. M.;Höfert, O.;Gotterbarm, K.;Lorenz, M. P. A.;Viñes, F.;Papp, C.;Görling, A.; Steinrück, H.-P. Graphene on Ni(111): Coexistence of Different Surface Structures. *J. Phys. Chem. Lett.* **2011**, *2*, 759-764.
17. Zhang, W.;Nefedov, A.;Naboka, M.;Cao, L.; Wöll, C. Molecular orientation of terephthalic acid assembly on epitaxial graphene: NEXAFS and XPS study. *Phys. Chem. Chem. Phys.* **2012**, *14*, 10125-10131.
18. Preobrajenski, A. B.;Ng, M. L.;Vinogradov, A. S.; Mårtensson, N. Controlling graphene corrugation on lattice-mismatched substrates. *Phys. Rev. B* **2008**, *78*, 073401.
19. Jeong, H. K.;Noh, H. J.;Kim, J. Y.;Colakerol, L.;Glans, P. A.;Jin, M. H.;Smith, K. E.; Lee, Y. H. Comment on ``Near-Edge X-ray Absorption Fine-Structure Investigation of Graphene''. *Phys. Rev. Lett.* **2009**, *102*, 099701.
20. Voloshina, E. N.;Generalov, A.;Weser, M.;Böttcher, S.;Horn, K.; Yu, S. D. Structural and electronic properties of the graphene/Al/Ni(111) intercalation system. *New J. Phys.* **2011**, *13*, 113028.
21. Fowler, B.;Lucas, C. A.;Omer, A.;Wang, G.;Stamenković, V. R.; Marković, N. M. Segregation and stability at $Pt_3Ni(111)$ surfaces and $Pt_{75}Ni_{25}$ nanoparticles. *Electrochim. Acta* **2008**, *53*, 6076-6080.
22. Najafi, E.;Cruz, D. H.;Obst, M.;Hitchcock, A. P.;Douhard, B.;Pireaux, J.-J.; Felten, A. Polarization Dependence of the C 1s X-ray Absorption Spectra of Individual Multi-Walled Carbon Nanotubes. *Small* **2008**, *4*, 2279-2285.
23. Bittencourt, C.;Hitchock, A. P.;Ke, X.;Van Tendeloo, G.;Ewels, C. P.; Guttmann, P. X-ray absorption spectroscopy by full-field X-ray microscopy of a thin graphite flake: Imaging and electronic structure via the carbon K-edge. *Beilstein J. Nanotechnol.* **2012**, *3*, 345-350.
24. Fischer, D. A.;Wentzcovitch, R. M.;Carr, R. G.;Continenza, A.; Freeman, A. J. Graphitic interlayer states: A carbon *K* near-edge X-ray-absorption fine-structure study. *Phys. Rev. B* **1991**, *44*, 1427-1429.
25. Kim, Y. S.;Jeon, S. H.;Bostwick, A.;Rotenberg, E.;Ross, P. N.;Stamenković, V. R.;Marković, N. M.;Noh, T. W.;Han, S.; Mun, B. S. Role of Transition Metal in Fast Oxidation Reaction on the Pt_3TM (111) (TM = Ni, Co) Surfaces. *Adv. Energy Mater.* **2013**, *3*, 1257-1261.
26. Yu, Y.-J.;Zhao, Y.;Ryu, S.;Brus, L. E.;Kim, K. S.; Kim, P. Tuning the Graphene Work Function by Electric Field Effect. *Nano Lett.* **2009**, *9*, 3430-3434.
27. Wan, J.;Fang, G.;Yin, H.;Liu, X.;Liu, D.;Zhao, M.;Ke, W.;Tao, H.; Tang, Z. Pt–Ni Alloy Nanoparticles as Superior Counter Electrodes for Dye-Sensitized Solar Cells: Experimental and Theoretical Understanding. *Adv. Mater.* **2014**, *26*, 8101-8106.
28. Quiroga, M. A.;Cabeza, G. F.; Castellani, N. J. Structural and magnetic properties of Ni/Pt multilayers. *Appl. Surf. Sci.* **2007**, *254*, 355-359.
29. Giovannetti, G.;Khomyakov, P. A.;Brocks, G.;Karpan, V. M.;van den Brink, J.; Kelly, P. J. Doping Graphene with Metal Contacts. *Phys. Rev. Lett.* **2008**, *101*, 026803.
30. Politano, A.;de Juan, F.;Chiarello, G.; Fertig, H. A. Emergence of an Out-of-Plane Optical Phonon (ZO) Kohn Anomaly in Quasifreestanding Epitaxial Graphene. *Phys. Rev. Lett.* **2015**, *115*, 075504.
